# Factors Influencing Positive Birth Experiences of First-Time Mothers

**DOI:** 10.1155/2013/349124

**Published:** 2013-09-19

**Authors:** Lena Nilsson, Tina Thorsell, Elisabeth Hertfelt Wahn, Anette Ekström

**Affiliations:** ^1^School of Life Sciences, University of Skövde, Sweden; ^2^Department of Obstetrics and Gynecology, Skaraborg Hospital Skövde, Sweden

## Abstract

*Objectives*. The objective of this study was to describe first-time mothers' experiences and reflections of their first birth. *Study Design*. This study is a part of a larger study which was carried out in southwestern Sweden in 2008. A qualitative method with content analysis was chosen for this study. The unit of data was 14 written narratives from the first-time mothers. *Results*. The theme “*To be empowered increases first-time mothers' chances for a positive birth experience*” crossed over into all the three categories: “*To trust the body and to face the pain*,” “*Interaction between body and mind in giving birth,*” and “*Consistency of support*.” *Conclusion*. In order to feel confident in their first childbirth, the women wanted to be confirmed and seen as unique individuals by the professionals and their partner. If professionals responded to the individual woman's needs of support, the woman more often had a positive birth experience, even if the birth was protracted or with medical complications.

## 1. Introduction 

The health care around childbirth has recently been concentrating on complications and risks for mother and child. The obstetric outcome has been focused on more than women's experience [1]. Giving birth is one of the most important events in life, which is a highly individual experience. The experience of childbirth plays a major role in how first-time mothers will develop good self-esteem [2], positive feelings for the baby, and an easier adjustment to motherhood role [3, 4], and also future childbirth experiences [4]. In order to provide better individual support to women during childbirth, the health care providers are required to put more focus on psychosocial aspects, but without neglecting medical safety [5]. In a study by Waldenström et al., it was found that the 10th woman in Sweden is so affected by their fear and anxiety before the birth that they seek professional help [6]. Melender reported the following factors which women are afraid of during pregnancy and childbirth: fear of childbirth, caesarean section, mother and infant health, health care professionals' actions, and subsequent family life [7]. Causes of fear can be that women had been frightened by others' stories of different problems. Fear appeared in the form of stress and effects on daily life as to avoid pregnancy and childbirth and a desire to have a caesarean [7]. Berg et al. also stress women's unique needs of support during birth, such as to be seen as an individual and to have a trusting relationship [8]. It has also been shown that women want a sense of security and to feel involved in decisions affecting them, during the childbirth period [8, 9]. There is a complexity to provide adequate support during childbirth. Therefore support from the social network [10–13] and from the health professionals is of importance [1]. A trusting relationship can be obtained by good communication and proficient behavior. By providing a sense of control, the women can be supported and guided by the midwife, on their own terms [1] in both normal and high-risk contexts [13]. A good quality of the professional support can also promote women's feelings and relationship with the child [3, 11]. Health care around childbirth should be based on evidence; therefore further research is needed about women's experiences of childbirth in order to develop the care requested by the women [13]. During the last two decades, empowerment has been increasingly used in the midwifery context to strengthen the woman and her family [14]. It is of importance to empower women to become more involved in maternal and child healthcare [15, 16]. In order to involve mother, more knowledge is needed about their own experiences and reflections. The objective of this study was therefore to describe first-time mothers' experiences and reflections of their first birth.

## 2. Methods 

A qualitative design content analysis was chosen for this study. Written narratives were found to be the most suitable data collection method for the present study in order to catch the women's freely described experiences of their first-time giving birth. Content analysis is a stepwise process of categorization based on the expression of thoughts, feelings, and actions described throughout the text. The intention of the analytical process is to remain close to the words of the text and to elicit the contextual meanings. Content analysis can either be manifest or latent, depending on the depth and level of abstraction. Manifest content is about the actual text, while latent content describes what the text is talking about [17]. For this study, latent content analysis was chosen to identify specific meanings in the women's narratives about their experiences of their first birth and reflections of the received support. The stepwise process of categorization is presented under the heading “data generation and analysis.”

### 2.1. Study Site and Participants

This study was undertaken during February to April 2008 in a hospital labor ward in a southwestern county of Sweden. The county holds 280,000 inhabitants. In 2006, there were 2204 births to 914 first-time mothers. The inclusions criteria for taking part in the present study were Swedish speaking first-time mothers with a normal birth and not cared for by the authors of this study and had healthy infants at discharge from hospital. The midwives at the maternity ward asked women that fit the inclusion criteria to participate in the study. The first-time mothers were provided information about the study, its objectives, and the rights of the research participants orally by the midwives at the maternity ward, and also by a leaflet. The first-time mothers were asked to freely describe their experiences of their first-time of giving birth in at least one page. 

### 2.2. Data Generation and Analysis

Written narratives were obtained from fourteen first-time mothers, one to two weeks after childbirth. The written narratives were two to seven pages. Data collection and part of the analysis were carried out simultaneously to follow up on the issues that were emerging and to decide when they were reaching saturation [17]. 

The written narratives were transcribed and analyzed separately using content analysis [17]. The transcripts from the first-time mothers were scrutinized several times, discussed, compared, and validated by the authors. Familiarity with the text was achieved by repeated reading. Words and sentences containing information relevant to the research questions were identified as meaning units, which were condensed and coded. The codes were grouped into subcategories and then categories. Data were further analyzed by reading across the categories, searching for new associations and meanings in the data. In the final step, findings were discussed and reflected upon, taking the research issues into account, and an overall theme emerged [18].

### 2.3. Ethics Approval

The ethical principles of the Helsinki declaration [19] guided the study, according to the Ethical Review Board [20]. The clinical head of service for the hospital labor ward gave access to undertake this study. The clinical head and the mothers were given information about the study in both verbal and written formats. This information included the voluntary participation and that they could withdraw from the study at any time without having to provide a reason. Confidential handling of all data protected the mother's identities. The narratives are anonymous in order to protect the mothers' identities. When asking about experiences from childbirth unexpected feelings could arise in participants. However in Sweden within the healthcare system, access to psychological support is available for all parents, when needed, regardless of participation in research studies or not.

## 3. Results 

The written narratives of the first-time mothers experiences and reflections of their first birth are presented as one main theme *“To be empowered increases first-time mother's chances for a positive birth experience” *with three categories: *“To trust the body and to face the pain,” “Interaction between body and mind in giving birth,” *and* “Consistency of support” *(Table 1).

Each category and its subcategories are presented using direct quotations in a conversational format. A code number for each respondent is included after the quotation (W1–W14). 

### 3.1. To Trust the Body and to Face the Pain 

#### 3.1.1. Body Strength

The first-time mothers described that the body's strength was affected by the order to go into themselves, knowing that nothing else matters and to allow the body to work all by itself. First-time mothers' experiences to trust the body was positive and gave a sense of power. The body had command and there was no turning back. The body was like a big muscle, while it was a body working at the same time. Contractions were handled differently by crying or shouting out while others felt completely focused on breathing through the contractions to give birth.

*When I called the delivery ward the first-time, it was scary, almost like it was when I realized that now there is no turning back. Now it's my body that has the command (W8).*

*By now I was so inside myself and in giving birth that nothing else matters (W2).*

#### 3.1.2. Manage Pain

The first-time mothers described that the body's strength was affected by how they could manage and deal with the pain. It was a new painful experience that they had not been through before. At the beginning of labor, first-time mothers experienced alternative pain relief as relaxing and comfortable. First-time mothers who decided not to have medical pain relief, such as Epidural Anesthesia (EDA), before birth, but did so because of severe pain, could experience it as a defeat. Despite of this, these mothers experienced good effect of EDA. The first-time mothers described a feeling of weakness. The strength declined during the first stage in labor because it lasted long and was painful and difficult.

* When the contractions started well, I was convinced it was just the pre-contractions, for so very painful was it not! In all cases, not as I imagined (W11).*

*I felt sorry for EDA: and I saw it as a defeat (W1).*

*After a while, I felt I needed more pain relief and my EDA, God, what a relief (W14).*

### 3.2. Interaction between Body and Mind in Giving Birth

#### 3.2.1. Control

The first-time mothers described a feeling of strength to have control during childbirth, such as concentrating on taking one contraction at a time. Loss of control was described by some first-time mothers as worse than the pain. First-time mothers also describe that the pain relief could affect the feeling of control in a positive way.

*I had lost sense of time and concentrated on taking one contraction at a time (W13).*

*The pain I could take but do not the loss of control, that gave me so much anxiety (W6).*

*When the anesthetic had taken, I started to become myself again, felt I had control again (W8).*

#### 3.2.2. Satisfaction

Feelings of satisfaction were expressed when the birth was an experience that the first-time mothers did not want to be without, and when they were proud of their own effort. A rapid labor progress and professional support gave feelings of satisfaction.

*My experience of childbirth is only good except that it took so long. I had contractions for 34 hours and I thought I was a bit too long. I had three midwives and two midwifery and ALL of it is an experience, I would never want to be without (W8).*

#### 3.2.3. Patience

First-time mothers described the varying degrees of patience during childbirth. Little patience was experienced when giving birth did not go ahead, waiting for the staff to come or waiting for pain relief. Patience was needed when having to wait to have the vaginal rupture stitched after the birth.

* It took almost 1 hour before the anesthesiologist arrived, it was horrible having to wait so long (W10) or It felt like it took 10 seconds before I got my EDA (W6).*

*I felt the vaginal rupture, I was sewn with 15 stitches but it was no problem for me (W7). or *

*The first two hours after the birth when I was sewn together, they were not funny (W2).*

#### 3.2.4. Happiness

First-time mothers described a sense of strength to bear children, an experience without equal. A feeling of indescribable happiness occurred when the baby slipped out and the pain disappeared. They also described a sense of unreality that they had given birth, to have managed it as they never thought they could do and this gave them a feeling of power.

*I understood not what it was that they added up to my stomach for something. *

*The best thing was to get the little girl on my chest, but it is difficult to understand that we now have a child (W7).*

*Finally we got to meet our daughter (W6).*

### 3.3. Consistency of Emotional Support

#### 3.3.1. Create Trust

The first-time mothers described that the feeling of trust is influenced by the environment in the maternity ward, tolerant, and peaceful atmosphere and the personal chemistry between the first time mothers and the midwives. To feel safe at home was important if the first-time mothers could stay quietly at home in the beginning of the labor. If the first-time mothers felt nervousness and concern about being at home, she perceived insecurity. The first-time mothers described lower support from both the partner and the professionals when the birth was complicated.

* For me and my partner's great relief, it will submit a midwife who says that now is the shift change and she will take care of us. It felt good and I felt at once more secure (W2).*

* It ran a lot of people in the room. My partner could not stand there and support me and he seemed nervous and backed away from among the staff. As a result, I did not feel entirely safe (W10).*

#### 3.3.2. Presence

The first-time mothers described the presence of a midwife and the partner during childbirth as a positive feeling of support, it helped the first-time mothers to cope with the childbirth as well as they did. The health care professionals' personal characteristics and emotional expressions affect perception of support. The staff was perceived as happy, really cute, lovely, nice, calm, competent, caring, and safe most of the time, but sometimes also as harsh, stressed, irritable, strict, and smelled bad of smoke. A feeling of inadequate support was experienced when midwives were not present and did not help when the first-time mothers asked for it. If the first time mothers felt abandoned for a long time and not knowing what happened, it gave a sense of desperation.

*I felt such gratitude to my midwife, an assistant nurse and my partner of course, without them, I had not fixed it as good as I did (W7).*

* The midwife was very stressed and had to leave me all the time (W8).*

#### 3.3.3. Responsiveness

The first-time mothers described that there was a sense of responsiveness to be seen and heard of the professionals and in an opposite way a decreased sense of responsiveness occurred in some unexpected situations.

* Someone came in who actually talked to me (W7).*

*The midwife had the idea that I had to put me on my back. This I refused, I can not! Then I realized that she was mad at me and she grabbed me from one side and the nurse from the other and forced to turn to me. It was very painful and dramatic for me (W11).*

### 3.4. Individualized Support to Women During Labor Increases Their Chances for a Positive Birth Experience

The first-time mothers wanted to be confirmed and seen as unique individuals by the professionals at their first childbirth. If professionals responded to the individual woman's needs of emotional support, the woman more often had a positive birth experience, even if the birth was protracted or with medical complications. Inadequate support from the midwives could lead to negative birth experience. The first-time mothers could have a negative experience, although well supported by midwives, because of very severe pain or risk of medical complications. 

## 4. Discussion 

The results showed that individualized emotional support empowers the first-time mothers during their first birth and increases their chances for a positive birth experience, even if the birth was protracted or with medical complications. Inadequate support from the midwives could lead to a negative birth experience. The women could have a negative experience, although well supported by midwives, because of very severe pain or the risk of medical complications. An empowerment approach means that the health care professionals should provide mothers with the information, expertise, support, and skills they need to enable an interactive participation [15, 16]. Women's feeling of a positive birth experience and being empowered was due to a presence and trustful relationship with the midwife and the partner. 

The mothers described that the body's strength was affected by how they could manage and deal with the pain; a feeling of losing control could be worse than pain, but pain relief could affect the feeling of control in a positive way. If the mothers not planned to have medical pain relief, but did so because of severe pain, they experienced a defeat, whether the pain was relieved or if they experienced remained control. If the women in this study felt empowered, it affected their experience positively, unless medical interventions or not, which is in line with earlier research [15, 16]. These results are also strengthen by earlier studies, which describe that mothers experience varying degrees of pain, anxiety, and panic during birth [21] the mothers ability to manage this, influence their self-confidence [1]. The obstetric outcome has unfortunately been focused on medical complications more than women's experience, even at normal births [1]. Waldenström et al. observe that first-time mothers often have a positive birth experience when they feel involved in decisions, which leads to that they can handle the situation better than they expected before giving birth [22]. In some obstetric situations, prompt decisions are sometimes required by professionals, for example, change birth position to the baby's heartbeat is affected. In these situations, it may be difficult to reach the women with information, and they may therefore feel that they are not involved. 

The results showed that the first-time mothers who either had a fast delivery process or received a request support of midwives and the partners had a positive childbirth experience. This result is confirmed by previous research [8, 11, 12, 23, 24]. Even those who had a long birth process had a positive experience if they got good support from the midwives and the partners. This differs from Nystedt describing how a prolonged delivery gives a negative experience and an insufficient power to give birth [10]. Goodman et al. describe that a positive childbirth experience increases first-time mothers' self-confidence and leads to positive expectations for future childbirth experiences. Negative experiences can lead to women choosing caesarean section at the next delivery or abortion as a future desire [4]. Inadequate support from the professionals could lead to a negative birth experience where women felt abandoned, immobilized, and not prioritized by the professionals. 

Using content analysis, this study investigated first time mother's experiences of birth and reflections of receiving support during the first time of giving birth. Individual written narratives were chosen as the data collection method, which was able to catch the first-time mother's narratives. According to Willman et al., words are often the best way to describe people's experiences, both spoken or written [25], which could provide information about meaningful values, experiences, and reflections [17]. Throughout the study, different steps were considered to enhance the trustworthiness of the study [26]. The transcripts from the first-time mothers were read through several times and were discussed, compared, and validated by the authors. The analysis was conducted carefully, and it is reasonable to assume that the phenomena under study are described from different perspectives, which provides credibility to the study. The material consisted of 14 stories written by first-time mothers who have given birth to their first child, which was judged to give a sufficient amount of material to reach saturation. Since the first-time mothers were very willing to share their experiences of their first birth, a great amount of material was received to process. When first-time mothers wrote down their stories freely and openly, they showed profound descriptions of their experience and did not have to limit themselves to a survey or interview. Speziale Streubert and Carpenter describe the use of written narratives; it is important that the researcher is quite clear about what he would like participants to write about so that it meets the objective [27]. In this study, the aim was experienced clearly by the first-time mothers, due to their profound descriptions of their experiences and reflections of their first birth. 

A positive childbirth experience is an important goal of obstetric care where childbirth is defined as a normal life event, with outcomes defined as “A live, healthy mother and baby and satisfaction of individual needs” [4, 28]. Lundgren and Berg describe that there is no simple solution to give all women a positive childbirth experience [1]. Within maternity care, a work environment needs to be created that focuses on a daily basis to evaluate and discuss the daily issues in order to improve evidence-based maternity care, with a view to influencing health professionals' documents [1, 13, 29]. Ekström et al. indicate that a process-oriented education for professionals involved in pregnancy, childbirth, and infancy in providing support around childbirth changes health professionals' attitudes and behavior in a favorable way [30–32]. This also led to the health professionals becoming more supportive in their meetings with the mothers [31], and the mothers' feelings and relationship with the child was also reinforced [3]. When mothers feel that midwives respect them and listen to them as individuals, they will feel confident about the health care. This calls for more research that could illuminate both the health care professionals and the mothers' experience of empowerment in health care.

## 5. Clinical Implications

Evidence-based knowledge around childbirth shows the importance of seeing childbirth as a normal life event; however, the focus in recent years has been on medical interventions, which is not in conformity with the evidence. The goal of obstetric care should be a healthy mother and a healthy baby including a positive birth experience for the women, regardless of normality or complication. It is of importance that healthcare organization promotes an evidence-based approach around childbirth where health professionals have ability and skills to evidence base the care around childbirth. It is essential that these goals are met in order to individualize support to women during labor which increases her chances for a positive birth experience

## 6. Conclusion 

Women's feeling of being empowered was due to a presence and trustful relationship with the professionals and the partners. If the women felt empowered, it resulted in an increased ability to feel control, strength of the body, satisfaction and reassurance, and as a result of this a better ability to manage the pain which occurred. Inadequate support from the professionals could lead to a negative birth experience where women felt abandoned, immobilized, and not prioritized by the professionals.

## Figures and Tables

**Table 1:** Categories with subcategories and theme identified from narratives with first-time mothers.

Categories and subcategories	Theme
*To trust the body and to face the pain* Body strength **Manage pain *Interaction between body and mind in giving birth* Control Satisfaction Patience **Happiness *Consistency of support* Create trust Presence Responsiveness	To be empowered increases first-time mother's chances for a positive birth experience
